# Severe illness caused by *Rickettsia sibirica* subspecies *sibirica* BJ-90 infection, China

**DOI:** 10.1038/emi.2017.95

**Published:** 2017-11-29

**Authors:** Hao Li, Xue-Ying Fu, Jia-Fu Jiang, Rui-Xia Liu, Ran Li, Yuan-Chun Zheng, Wen-Jie Qi, Wei Liu, Wu-Chun Cao

**Affiliations:** 1 State Key Laboratory of Pathogen and Biosecurity, Beijing Institute of Microbiology and Epidemiology, Beijing 100071, China; 2 Beijing Friendship Hospital, Capital Medical University, Beijing 100050, China; 3 Beijing Chao-Yang Hospital, Beijing Institute of Respiratory Medicine, Capital Medical University, Beijing 100020, China; 4 Mudanjiang Forestry Central Hospital, Mudanjiang 157000, China

**Dear Editor,**

Human infection of *Rickettsia sibirica* subsp. *sibirica* was first described in Russian Far East in the 1930s.^1^ Since then, this spotted fever group (SFG) rickettsiosis has been found across a large territory of north Asia, including Russia, China, and Mongolia and Kazakhstan.^2, 3, 4, 5^ Common clinical manifestations include fever, headache, weakness, myalgia, rash, eschar, and lymphadenopathy. The disease is usually mild, rarely related to severe complications, and nonfatal.

*R. sibirica* subsp. *sibirica* BJ-90, a variant of *R. sibirica*, was first isolated from *Dermacentor sinicus* in China in 1990,^6, 7^ and has recently been detected in a patient with multiorgan dysfunction.^8^ Here we report two cases with severe illness caused by *R. sibirica* subsp. *sibirica* BJ-90 in northern China.

An 81-year-old woman from Harbin City was admitted to Mudanjiang Forestry Central Hospital in northern China on June 2013 (Supplementary Figure S1), with fever (temperature, up to 39.0 °C), fatigue, myalgias, and arthralgia for 7 days, and a hyperpigmented maculopapular rash for 3 days (Figure 1). She reported having a tick bite at the waist when taking a walk in a public park 3 days before illness onset. During the third days of symptoms, she received treatment with cephalosporin and ibuprofen, but her condition did not improve. On admission, blood test abnormalities included: leukocytosis (white blood cell count, 18.1 × 10^9^/L), thrombocytopenia (platelet count, 49 × 10^12^/L), neutrocytosis (neutrophil count, 13.8 × 10^9^/L); increased levels of liver enzymes (alanine aminotransferase, 73 U/L; aspartate aminotransferase, 86 U/L), glutamyl transpeptidase (95 U/L), creatinine (525 μmol/L), urea nitrogen (44.2 mmol/L), uric acid (648 μmol/L), high-sensitivity C-reactive protein (72 mg/L), and β2-macroglobulin (25 mg/L); decreased levels of albumin (26.0 g/L), cholinesterase (3,192 U/L), calcium (1.6 mmol/L), and carbon dioxide combining power (15 mmol/L). Urine tests showed an elevated level of erythrocytes (80 cells per μL) and total protein (0.3 g/L). Chest radiograph showed increased lung markings, and ultrasonic examination revealed cholecystitis, and hepatic and renal diffuse changes. The patient was intravenously treated with doxycycline based on the clinical symptoms and the preceding tick bite. 2 days after admission, the patient’s leukocytosis, thrombocytopenia, neutrocytosis, blood biochemistry abnormalities, and hepatic and renal dysfunction did not improve (Supplementary Table S1); her condition deteriorated with oliguria, chest distress, rising heart rate, and oedema legs. The patient was subsequently administered with respirator and supportive treatment, but she deteriorated further and developed coma. The patient died 5 days after hospitalization.

A 62-year-old woman from Beijing City was admitted to Beijing Chao-Yang Hospital in northern China on June 2016 (Supplementary Table S1), with fever (temperature, up to 39.0 °C), fatigue, dizziness, tinnitus, and chest distress for 6 days, and a sporadic petechial rash for 2 days (Figure 1). The patient reported that she noted a tick bite at the left armpit 2 days before illness onset. The laboratory findings on admission were as follows: leukopenia (white blood cell count, 3.7 × 10^9^/L), thrombocytopenia (platelet count, 82 × 10^12^/L), lymphopenia (lymphocyte count, 0.4 × 10^9^/L); increased levels of liver enzymes (alanine aminotransferase, 220 U/L; aspartate aminotransferase, 386 U/L), lactate dehydrogenase (772 U/L), hydroxybutyrate dehydrogenase (557 U/L), C-reactive protein (78 mg/L), and D-Dimer (6.31 mg/L); and decreased levels of albumin (34.8 g/L). Urine tests showed an elevated level of erythrocytes (10 cells/μL) and total protein (0.25 g/L). Computed tomography showed thickening of left pleura and slight hydropericardium, and ultrasonic examination revealed cyst of right kidney. 3 days later, she developed oedema legs. After treated with oral minocycline for 7 days, the patient’s clinical manifestations and laboratory abnormalities resolved, except slightly increased levels of lactate dehydrogenase and hepatic aminotransferases.

Because the patients presented with typical rash and had a recent history of tick bite, SFG rickettsiae was considered as the causative agent. 0.2 mL EDTA-anticoagulant blood samples were obtained from the patients before initiation of therapy, and DNA was extracted with the Blood Mini Kit (Qiagen, Germantown, USA) according to the manufacturer’s instructions. A nested PCR targeting the citrate synthase gene (*gltA*) was performed as previously described.^9^ For further confirmation, the outer membrane protein A gene (*ompA*) was amplified using external primers Rr190.70p and Rr190.602n,^10^ and internal primers 190.70-38s1M (5′-AAA GCC GCT TTA TTC ACC-3′) and 190.602-384r1M (5′-GGC AAC AAG TTA CCT CCC-3′) modified according to previous study.^7^ To obtain nearly entire length of both *gltA* and *ompA* genes, hemi-nested PCR assays were performed using the product of the first PCR round as templates. PCR fragments were sequenced on a 3730 DNA sequencer (Applied Biosystems, Foster City, CA, USA) and aligned by using ClustalW software.

Nucleotide sequences of both *glt*A (1290 bp) and *omp*A (533 bp) genes (GenBank accession numbers KY617774 and KY617775 for Case 1; KM288711 and KM288712 for Case 2) from the two patients were identical. Sequence analyses showed that the two gene sequences had 100% similarities to the corresponding sequences from *Rickettsia sibirica* subsp. *sibirica* BJ-90 (GenBank accession numbers JX945526 and AF179365, respectively).

Serum samples were tested by indirect immunofluorescence assay (IFA) for IgM and IgG against *R. rickettsii* by using a commercially IFA kit (Focus Diagnostics Inc., Cypress, USA). For Case 1, the serum sample collected at admission was available, and IgM and IgG titers were 256 and 512, respectively. For Case 2, IgM titers of 128 and IgG titers of 256 were observed in the serum sample obtained at admission, and IgM titers of 256 and IgG titers of 1024 were observed in the serum sample obtained 14 days after illness onset. In addition, PCR and IFA results for other tick-borne pathogens, including *Anaplasma phagocytophilum*, *Babesia microti*, *Borrelia burgdorferi* sensu lato, severe fever with thrombocytopenia syndrome bunyavirus, and tick-borne encephalitis virus, were negative.

In the current study, two cases of *Rickettsia sibirica* subsp. *sibirica* BJ-90 infection, including one fatal case, were identified. Both patients presented with severe clinical manifestations, including a spread rash around the trunk and extremities, chest distress, oedema legs, and multiorgan dysfunction. Moreover, one patient developed oliguria and coma, and died 12 days after illness onset. The fatal outcome may be associated with several factors, among which are old age, late start of effective treatment, and early laboratory abnormalities such as liver dysfunction, acute renal failure, hypoalbuminemia, and hypocalcemia. Based on the clinical data from our two patients and the previously reported patient,^8^ we suppose a high pathogenicity of *Rickettsia sibirica* subsp. *sibirica* BJ-90, which needs to be corroborated by further investigations from a large number of cases.

*R. sibirica* subsp. *sibirica* BJ-90 have been detected from *D. sinicus* in China and from *D. silvarum* in Russia,^6, 11^ indicating that *Dermacentor* ticks are potential vectors for the pathogen. Considering the wide distribution of *Dermacentor* ticks in northern China, it is deduced that the health burden of human infection with *Rickettsia sibirica* subsp. *sibirica* BJ-90 might be beyond our understanding, although only three cases have been reported until now. Extended investigation and case surveillance are required to understand the distribution of human infection with *Rickettsia sibirica* subsp. *sibirica* BJ-90.

Diagnosis of tick-borne SFG rickettsioses is confirmed almost exclusively by serologic methods in China,^12^ which is an effective tool but cannot identify the causative agent to species level. In northern China where complex circumstance of SFG rickettsiae is endemic, clinicians should be aware of the potential high pathogenicity of *R. sibirica* subspecies *sibirica* BJ-90, and molecular-based diagnostic approaches should be applied on suspected patients.

## Figures and Tables

**Figure 1:**
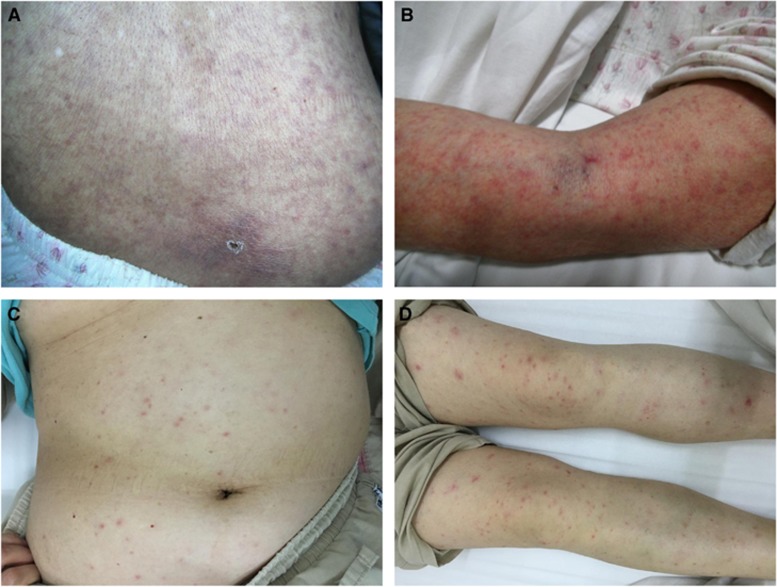
Typical signs of the two patients with *Rickettsia sibirica* subspecies *sibirica* BJ-90 infection. Case 1: (**A**) shows a crusted eschar at the site of tick bite, and a maculopapular, hyperpigmented, non-itchy rash affecting the trunk; (**B**) shows a maculopapular, red, non-itchy rash on the arms. Case 2: (**C**) shows a sporadic petechial rash affecting the trunk; (**D**) shows a red petechial rash on the legs.
