# Supplementary material for: Severe illness caused by Rickettsia sibirica subspecies sibirica BJ-90 infection, China
Source: Emerg Microbes Infect. 2017 Nov 29;6(11):e107–. doi: 10.1038/emi.2017.95 (PMC5717096; doi:10.1038/emi.2017.95)
Supplement: Supplementary Table S1 [file emi201795x1.docx]

**Supplement Table T1** Laboratory test results of Case one, the fatal patient.

| **Characteristic** | **On admission**  **6/17/2013** | **Duration of hospital stay**  **6/19/2013** | **Normal range** |
| --- | --- | --- | --- |
| White-cell count (×10^9^/L) | 18.1 | 21.7 | 4.0–10.0 |
| Platelet count (×10^9^/L) | 49 | 86 | 150–300 |
| Lymphocyte count (×10^9^/L) | 3.5 | 5.6 | 0.8–4.0 |
| Neutrophil count (×10^9^/L) | 13.8 | 14.7 | 2.0–7.0 |
| Erythrocyte count (×10^12^/L) | 4.3 | 3.8 | 3.8–5.2 |
| Hematocrit (%) | 36.6 | 33.3 | 33.5–45.0 |
| Hemoglobin (g/L) | 138 | 120 | 115–150 |
| Albumin (g/L) | 26 | 26 | 35–55 |
| Total bilirubin (µmol/L) | 19 | 15 | 5–21 |
| Alanine aminotransferase (U/L) | 73 | 59 | 5–40 |
| Aspartate aminotransferase (U/L) | 86 | 70 | 8–40 |
| Glutamyl transpeptidase (U/L) | 95 | 92 | 8–30 |
| Alkaline phosphatase (U/L) | 116 | 250 | 45–128 |
| Cholinesterase (U/L) | 3 192 | 3 065 | 4000–12 600 |
| Creatinine (µmol/L) | 525 | 469 | 45–84 |
| Urea nitrogen (mmol/L) | 44.2 | 43.5 | 3.0–8.2 |
| Uric acid (µmol/L) | 648 | 797 | 150–350 |
| Glucose (mmol/L) | 5.1 | 3.5 | 3.8–6.1 |
| Kalium (mmol/L) | 4.3 | 3.9 | 3.5–5.5 |
| Sodium (mmol/L) | 133 | 138 | 135–145 |
| Chlorine (mmol/L) | 95 | 97 | 96–106 |
| Calcium (mmol/L) | 1.6 | 1.6 | 2.1–2.9 |
| Carbon dioxide combining power (mmol/L) | 15 | 19 | 21–29 |
| High-sensitivity C-reactive protein (mg/L) | 72 | 66 | 0–3 |
| β2- macroglobulin (mg/L) | 25 | 25 | 0–3 |
| Total cholesterol (mmol/L) | 4.1 | 4.2 | 0–5.2 |
| Triglyceride (mmol/L) | 5.7 | 5.2 | 0.6–1.9 |
