# Supplementary figures and images for: Severe illness caused by Rickettsia sibirica subspecies sibirica BJ-90 infection, China
Source: Emerg Microbes Infect. 2017 Nov 29;6(11):e107–. doi: 10.1038/emi.2017.95 (PMC5717096; doi:10.1038/emi.2017.95)

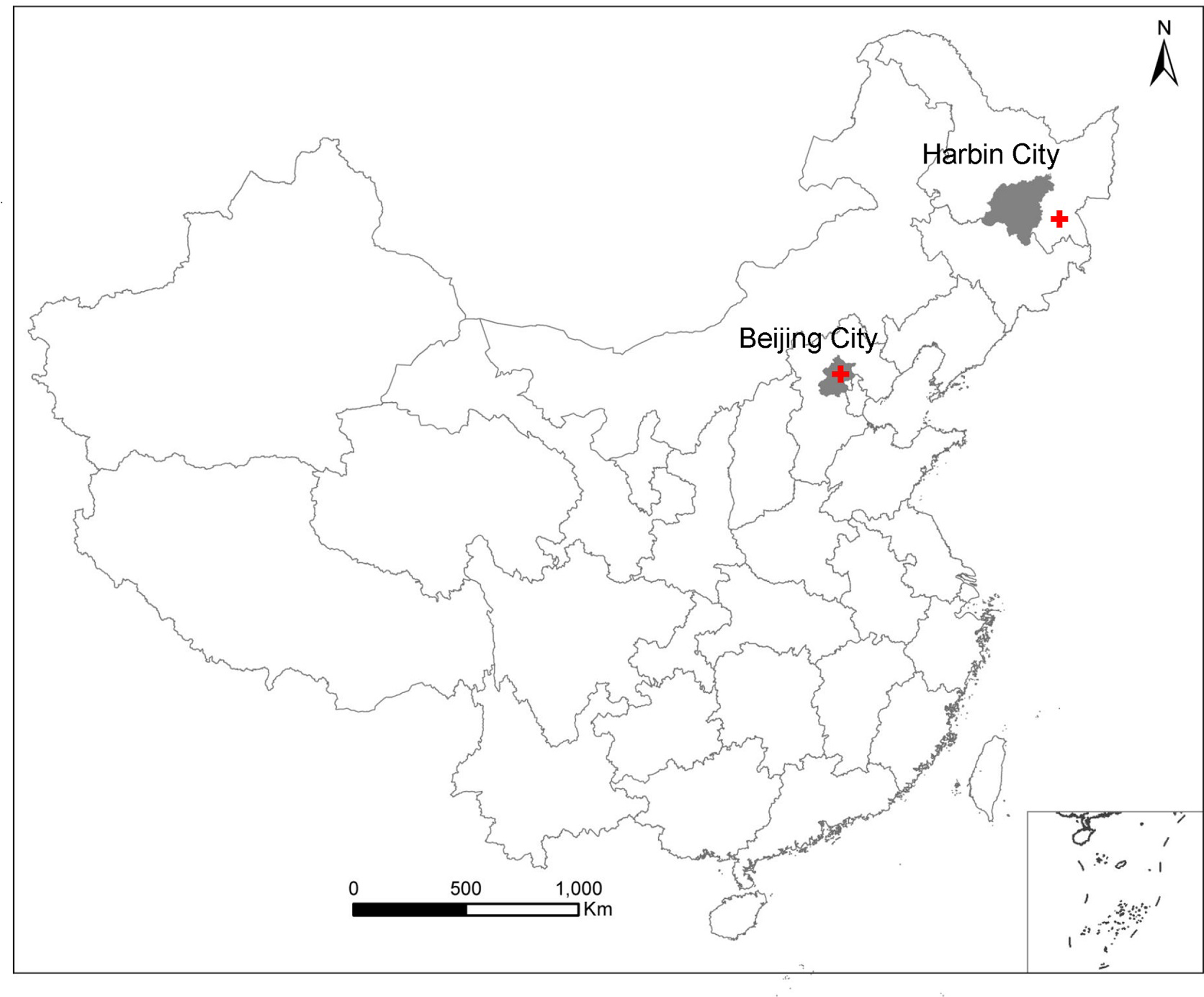

Supplement: Supplementary Figure S1 [file emi201795x2.tif]
